# Review of Supramolecular Oleogel Lubricants

**DOI:** 10.3390/gels12040338

**Published:** 2026-04-17

**Authors:** Lei Wei, Minghui Xiong, Haoye Wang, Yuelin Chen, Song Chen, Jiaming Liu

**Affiliations:** College of Mechanical Engineering, Hunan Institute of Science and Technology, Yueyang 414006, China

**Keywords:** supramolecular oleogel lubricant, green lubrication, intelligent lubrication, nanocomposite, extreme operating conditions

## Abstract

Supramolecular oleogel lubricants construct a three-dimensional network structure within base oils through gelator-mediated non-covalent interactions, such as hydrogen bonding, van der Waals forces, and π–π stacking. These materials demonstrate unique advantages in mitigating issues inherent to traditional lubricants, including leakage, volatility, creep, and poor heat dissipation. Focusing on structural design and performance regulation, this review systematically summarizes the current development of supramolecular oleogel lubricants in the fields of green lubrication, extreme operating conditions, and nanocomposite lubrication. Specifically, it outlines the structure-property relationships between gelators and base oils in green lubrication systems, and elucidates the applications in radiation-resistant, high-load-bearing, and intelligently responsive lubrication. Strategies for utilizing nanocomposite supramolecular oleogels to resolve nanomaterial dispersion challenges are discussed, and the latest advancements in engineering applications are illustrated. By summarizing the development of supramolecular oleogel materials, this work can provide theoretical references for the future design and preparation of these lubricants.

## 1. Introduction

Friction and wear are widespread in mechanical equipment across industries such as automotive, shipping, and aerospace [1], acting as primary factors leading to energy loss, component failure, and increased maintenance costs [2]. Statistically, approximately 23% of global energy consumption originates from frictional contact losses, with 20% attributed to friction and 3% related to wear [3]. Lubricating oils and greases are the two most common lubricating materials for mitigating friction; however, under complex and extreme conditions, their respective limitations become increasingly apparent. While lubricating oils possess excellent fluidity and easily form lubricating films, they struggle to remain at the friction interface for long periods, making them highly susceptible to leakage and splashing. Reportedly, about 40–50% of commercial lubricants eventually enter the environment through leakage [4], which not only compromises long-term lubrication performance but also causes severe environmental pollution [5]. In contrast, traditional lubricating greases, composed of base oils and thickeners [6], exhibit excellent interfacial retention and anti-leakage capabilities due to their high viscosity. However, greases rely on irreversible physical entanglement networks and are prone to permanent structural damage [7]. Lacking self-healing capability and suffering from poor heat dissipation, they ultimately lead to lubrication failure. Therefore, developing a new generation of lubricating materials that combines the fluidity of oils with the retention capability of greases has become a key challenge in enhancing lubrication [8,9].

In recent years, the development of supramolecular gel lubricants has provided a new approach for designing high-performance lubricating materials [10]. These materials rely on the self-assembly of gelators to capture liquid solvents, enabling a gel-sol state transition under specific conditions. The self-assembly of supramolecular gels is driven by non-covalent interactions, such as hydrogen bonding, π–π stacking, and van der Waals forces, to form a three-dimensional network structure [11]. Although supramolecular gel lubricants show immense application potential, existing research predominantly focuses on hydrogel lubrication systems, mainly applied in fields such as soft material interfacial friction, biolubrication, and biomimetic joints [12,13,14]. Mechanistically, hydrogels rely on high water content and hydration layers to achieve low friction; under low contact stresses (e.g., loads ≤ 30 N), their friction coefficient is reduced to below 0.02 [15,16]. However, constrained by their aqueous solvents, hydrogels possess fatal flaws under extreme conditions, including high-temperature evaporation, low-temperature freezing, and the tendency to corrode metallic friction interfaces [17]. Furthermore, under high loads, the gel network is easily torn, causing the instant depletion of water and resulting in lubrication failure. Additionally, an inherent contradiction exists between their mechanical and lubricating properties [18]; a high water content weakens intermolecular interactions and loosens the network to promote lubrication, but simultaneously causes a decline in mechanical properties [19]. Consequently, current hydrogel lubricants struggle to meet the lubrication demands of mechanical equipment under extreme conditions, limiting their application scope [20]. In contrast, supramolecular oleogel lubricants, utilizing oil as the solvent, are more suitable for extreme conditions. Their core advantage lies in excellent thixotropy and dynamic reversibility: under shear force, the gel network dissociates (gel-sol phase transition) to release the base oil, which exhibits strong environmental adaptability and provides highly efficient lubrication; once the shear stops, the network rapidly reorganizes to restore the gel state, thereby firmly locking the oil and retaining it at the interface. This characteristic perfectly overcomes the dual defects of liquid leakage and irreversible structural damage in greases [21]. Even under extreme loads ranging from hundreds to thousands of Newtons, the friction coefficient can be reduced by approximately 0.1 or even lower, demonstrating extraordinary developmental prospects [22,23]. Furthermore, supramolecular oleogel lubricants are highly designable; their tribological performance can be further enhanced through strategies such as the molecular structure design of gelators and nanomaterial doping [24]. However, systematic review studies focusing on supramolecular oleogel lubricants remain relatively scarce. Existing reviews have primarily explored the relationships between the molecular structure of gelators and the gelation, rheology, and tribological properties of gel lubricants [25]. Moreover, these existing reviews mix hydrogel and oleogel systems in their discussions, leaving a blank in systematic and in-depth reviews dedicated specifically to supramolecular oleogel lubricants. This review investigates green lubrication in detail, a topic untouched by predecessors. Secondly, in discussing extreme conditions, this review fills the gap regarding the more critical enhancements in ultra-high load-bearing capacity and smart responsive lubrication design. Additionally, this review conducts a detailed study on the strategies and mechanisms by which nanocomposite supramolecular oleogels resolve the long-term dispersion stability of nanomaterials.

Based on this, this review systematically summarizes the latest research progress on supramolecular oleogel lubricants in recent years and constructs an analytical framework from four interrelated dimensions: (1) green oleogel Lubricants: Discusses the sustainable green development of lubrication systems from several perspectives, including intrinsic green lubrication performance, gelator design, base oil substitution, and green synthesis processes; (2) functionalized Supramolecular oleogel Lubricants for Extreme Conditions: Introduces functional group modifications, the development of polymer or dual-component oleogel, and smart lubrication designs, imparting lubrication materials with radiation resistance, high-load bearing, and adaptive lubrication properties, thereby enhancing their reliability and lubrication performance under extreme working conditions; (3) nano-Composite Supramolecular oleogel Lubricants: Analyzes the synergistic effect between nanomaterials and supramolecular gels, focusing on solving the aggregation issue of nanomaterials in traditional liquid lubricants, improving dispersion stability and tribological performance; (4) practical Applications and Development of Supramolecular oleogel Lubricants in Engineering: Systematically reviews the exploration of supramolecular oleogel lubricants in composite systems, marine drag reduction, antifouling, and other engineering scenarios, with a focus on the advantages of solid lubricating films combined with supramolecular oleogel lubricants in enhancing lubrication reliability under extreme environments. This review aims to reveal the evolutionary development trends of supramolecular oleogel lubricants and provide insights for the design of future lubrication materials.

## 2. Green Supramolecular Oleogel Lubricants

The self-assembled three-dimensional network structure of supramolecular oleogels endows lubricating materials with excellent creep recovery, thixotropy, and tribological properties. While ensuring superior lubrication performance, this approach achieves effective physical confinement of base oils, mitigates leakage risks, and represents a cutting-edge strategy for realizing green lubricant performance. Xie et al. [26] developed a series of perfluorinated supramolecular oleogel lubricants (PFG) using PAO10 as the base oil, which exhibited outstanding thixotropic properties, creep recovery performance, and exceptional tribological characteristics. The study demonstrated that under high shear rates, the three-dimensional gel network structure of the lubricant is disrupted, resulting in viscosity decreased to 0.2 Pa s and rapid disintegration through gel-sol transition, thereby releasing the base oil at the friction interface to provide lubrication. Upon low shear or cessation of shearing (increases to more than 2 Pa s), PFG rapidly self-reassembles to restore its gel structure within a short period, effectively preventing base oil leakage and volatilization during equipment shutdown or low-speed operation. This exceptional green lubrication performance demonstrates advantages that conventional lubricants do not possess. Supramolecular oleogel lubricants have also exhibited outstanding green lubrication performance in various other base oils. Zhang et al. [27] employed pyromellitic acid derivative (BHTA) as a low-molecular-weight gelator to successfully prepare supramolecular oleogel lubricants in n-hexadecane, diesel, liquid paraffin, and multiple industrial base oils (including 150BS, 500SN, PAO10, and PAO40). All these lubricants demonstrated favorable thixotropic properties, creep recovery performance, and tribological characteristics. Molecular simulations revealed that the lubricants primarily rely on hydrogen bonding interactions between carboxyl and amide groups of BHTA molecules to self-assemble into three-dimensional network structures that capture and immobilize the base oil, thereby inhibiting leakage. However, the validation of creep recovery and thixotropy in most studies mainly relies on short-term rheological tests involving only a few shear cycles (typically 3 to 5). Although these tests demonstrate that gelators like PFG and BHTA possess rapid sol-gel transition capabilities, they are insufficient to simulate the continuous and highly repetitive mechanical shear experienced in actual long-term operation. Under prolonged cyclic shear, dynamic non-covalent networks (such as hydrogen bonds) may suffer from structural fatigue or permanent degradation. Whether these supramolecular networks can maintain a consistent recovery rate over millions of working cycles, thereby continuously achieving anti-leakage green performance, remains an unverified limitation.

**Figure 1:**
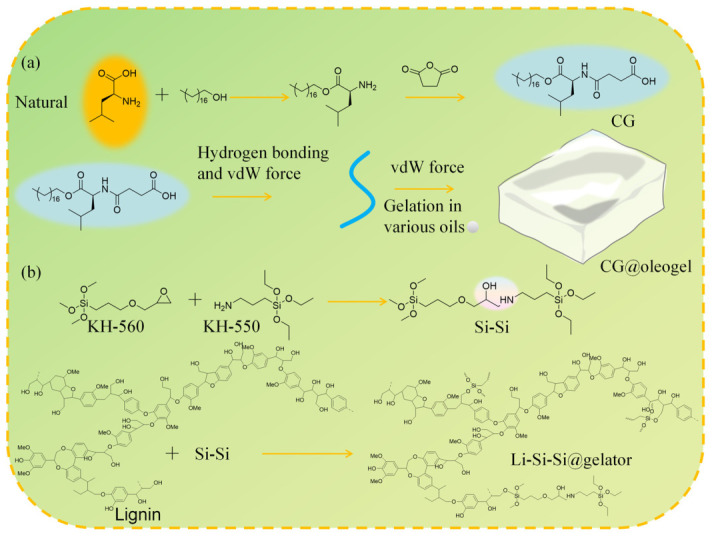
(**a**) Schematic illustrations for preparation process of eco-friendly gelator CG and self-assembled process, and (**b**) reaction schematic diagram of Li–Si–Si@gelator.

Beyond the inherent green performance of supramolecular oleogel lubricants in suppressing base oil leakage through physical state transitions, the chemical origins of both gelators and base oils fundamentally determine the environmental friendliness of lubricants. Currently, researchers have developed gelators from natural renewable molecules or substituted mineral oils with vegetable oils, which has emerged as a core strategy for developing green supramolecular oleogel lubricating materials. Qi et al. [28] designed a class of terminal carboxyl-functionalized gelators using natural amino acids as raw materials to construct environmentally friendly supramolecular oleogel lubricants, as shown in Figure 1a. Their excellent lubrication performance originates from a dual-layer friction reduction mechanism synergistically established by the physical gel oil film and tribochemical film: the terminal carboxyl groups anchor to metal surfaces to stabilize the boundary oil film, while friction-induced interfacial reactions generate dense tribochemical films in situ, significantly enhancing oil film stability and anti-wear/friction-reducing characteristics. This work further systematically revealed the influence of base oil viscosity on gel structure: base oils with moderate viscosity and abundant branched alkanes facilitate the formation of uniform and moderately entangled fiber network structures, thereby achieving a balance between mechanical strength and lubrication performance. Furthermore, Gao et al. [29] constructed supramolecular oleogel lubricants using modified cellulose nanofiber (CNF) gels, which enhanced tribological performance while maintaining the renewable, low-toxicity, and bio-friendly characteristics of CNF, demonstrating the application potential of bio-based gelators in green lubrication. Regarding environmentally friendly alternatives for base oils, vegetable oils have become ideal substitutes for petroleum-based base oils owing to their inherent biodegradability. Tian et al. [30] developed supramolecular oleogel lubricants using various vegetable oils (soybean oil, rapeseed oil, sunflower oil, and cottonseed oil) as base oils to replace petrochemical-based counterparts. Tribological tests demonstrated that these lubricants retained the excellent tribological performance of the original vegetable oils, and their oxidative stability was enhanced through the addition of natural antioxidants, addressing the inherent limitation of oxidation susceptibility in vegetable oils. Martín-Alfonso et al. [31] formulated various supramolecular oleogel lubricants based on 30 wt.% montmorillonite and different vegetable oils (olive oil, castor oil, soybean oil, linseed oil, and sunflower oil). Tribological performance tests revealed differences in the ability of various vegetable oils to form boundary lubricating films at friction interfaces, with olive oil, sunflower oil, and linseed oil formulations exhibiting more stable interfacial lubricating films and superior lubrication performance. Martín-Alfonso et al. [21] also discovered that the fatty acid composition and content in vegetable oils exert a decisive influence on gel network strength: higher contents of saturated fatty acids and monounsaturated fatty acids combined with lower contents of unsaturated and polyunsaturated fatty acids are more conducive to constructing dense and robust three-dimensional network structures, thereby forming more stable interfacial lubricating oil films. Furthermore, researchers have combined bio-based gelators with vegetable oil bases to construct fully bio-based supramolecular oleogel lubricants, achieving complete greening of lubricants. Wu et al. [32] proposed a gelator design strategy entirely derived from biomass feedstocks, synthesizing a lignin-based gelator from lignin, malic acid, and epoxidized soybean oil to construct a renewable and biodegradable supramolecular oleogel lubricant system. Studies demonstrated that this gelator significantly enhanced lubrication performance when combined with castor oil or epoxidized soybean oil, which may be attributed to the participation of lignin structural units in interfacial film construction, improving film stability. It should be noted that this system exhibits pronounced selectivity toward base oil polarity, capable of establishing stable networks only in vegetable oils containing polar functional groups such as hydroxyl or epoxy groups, which limits its universality in non-polar mineral oils and PAO-type base oils. Also utilizing lignin, Wu et al. [33] modified organosolv lignin to construct a castor oil-based supramolecular oleogel lubricant system, as shown in Figure 1b. This system ingeniously exploited the abundant hindered phenolic hydroxyl groups in modified lignin as free radical scavengers, effectively suppressing the chain reactions of lipid radicals and inhibiting oxidative degradation of the oil phase during lubrication. This approach not only achieved excellent green lubrication performance but also simultaneously resolved the oxidation susceptibility problem of vegetable oils, providing a new paradigm for developing high-performance, multifunctional green lubricating materials. Despite the innovations achieved in the use of bio-based gelators and vegetable oils, there is also a fundamental polarity mismatch problem; many green gelators naturally contain abundant polar functional groups, resulting in their inability to self-assemble into stable gel networks within non-polar base oils. Given that non-polar mineral oils and synthetic polyalphaolefins (PAOs) still occupy an absolute dominant position in the mainstream industrial lubricant market, this specific compatibility severely limits the universal applicability of green gelators. Secondly, the inherent oxidative instability of vegetable oils remains an unresolved challenge in practice. Current strategies involve the external addition of antioxidants or relying on the specific free-radical scavenging structures of certain gelators (such as modified lignin), but this is highly system-specific and cannot be universally promoted across different gel systems. Furthermore, the structural strength of the gel network is highly sensitive to the specific fatty acid distribution in vegetable oils. Because the composition and content of saturated and unsaturated fatty acids vary significantly among different plant sources, generalizing vegetable oils as a uniform base oil is fundamentally flawed. Future research requires a more detailed investigation into how the fatty acid chain lengths and degrees of unsaturation in different vegetable oils affect the self-assembly of the gel network.

**Figure 2:**
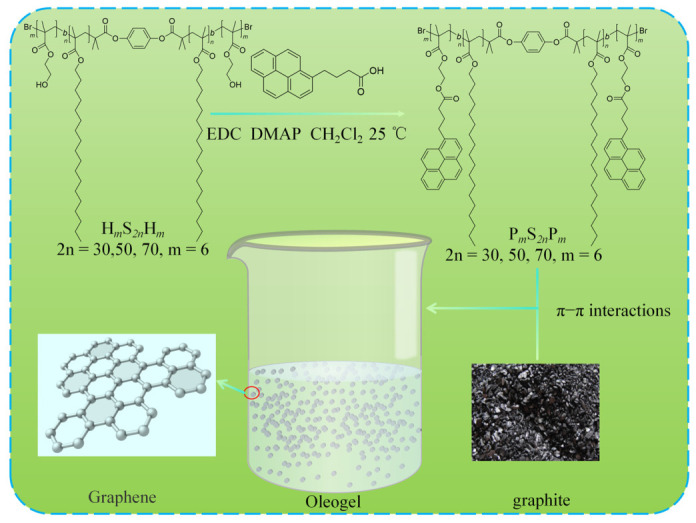
Schematic diagram of the preparation process of green supramolecular oleogel lubricant.

The evaluation system for green lubricating materials encompasses not only the biodegradability and ecological compatibility of the materials themselves, but also the preparation processes, which constitute a critical dimension determining their sustainable development. Zhang et al. [34] prepared an N-octadecyl-D-gluconamide (NOG) gelator following the principles of non-toxic reactants and environmentally friendly processes. In paraffin, pentaerythritol oleate, and polyethylene glycol oil, this gelator self-assembles solely through hydrogen bonding interactions between hydroxyl and carbonyl groups in NOG molecules. This supramolecular oleogel lubricant exhibited excellent friction-reducing and anti-wear characteristics in rheological tests. Similarly, addressing the complex and environmentally burdensome issues associated with the preparation of graphene and its derivative additives, such as strong acid oxidation and tedious washing procedures, Liang et al. [35] introduced a polymer gelator containing pyrene groups. By exploiting the π–π stacking interactions between pyrene and graphite layers, this approach induced in-situ exfoliation of graphite in base oil while simultaneously constructing a three-dimensional gel network. This strategy bypasses the conventional strong oxidation-reduction exfoliation route, operates under mild reaction conditions, and reduces costs, process complexity, and toxic waste effluent discharge, while achieving the incorporation of graphite materials into supramolecular oleogel lubricants to enhance tribological performance, thereby developing a low-cost, green, and high-performance lubrication methodology, as shown in Figure 2. In summary, the development of supramolecular oleogel lubricants is establishing a multi-dimensional green lubrication system: during the service stage, their inherent green performance effectively suppresses lubricant leakage at the physical level; in terms of raw material selection, the introduction of natural renewable gelators and vegetable oil bases achieves biodegradation and environmental compatibility; in the manufacturing process, the mild characteristics of supramolecular self-assembly establish a low-energy-consumption and pollution-free green preparation methodology. This integrated solution strategy, combining physical leakage prevention, raw material renewability, and green processing, fully demonstrates the tremendous application potential and development prospects of supramolecular oleogel lubricants in the field of sustainable tribology.

## 3. Functionalized Supramolecular Oleogel Lubricants for Extreme Conditions

**Figure 3:**
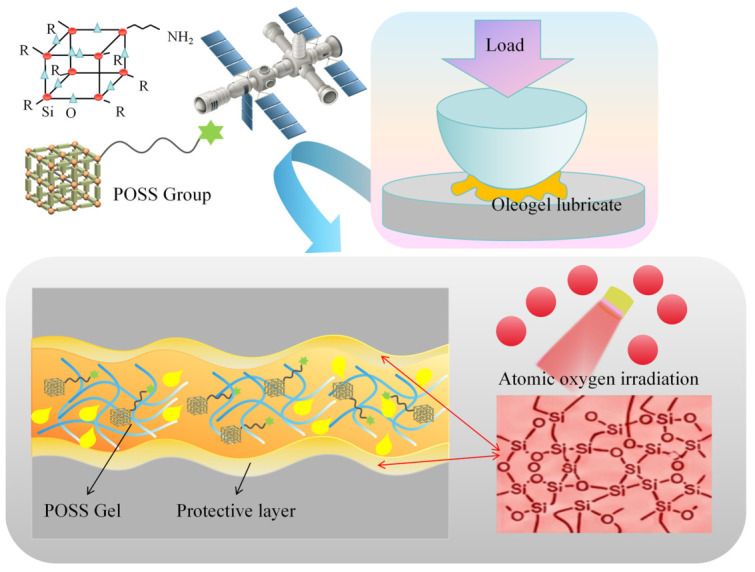
Schematic diagram of POSS oleogel lubricant against atomic oxygen Radiation.

In space radiation environments, mechanical systems are subject to the influence of atomic oxygen (AO) irradiation factors, imposing zero-failure requirements on the lubrication reliability of mechanical moving components in space environments. Based on the stable three-dimensional network characteristics of supramolecular gels and through molecular structure design to introduce functional groups, it is promising to develop novel lubricating materials with high-performance lubrication, radiation resistance, and anti-creep properties for space applications. Yu et al. [36] achieved enhanced resistance to atomic oxygen irradiation by introducing polyhedral oligomeric silsesquioxane (POSS) groups into 12-hydroxystearic acid gelators, and subsequently constructed a supramolecular oleogel lubricant using multiply alkylated cyclopentane as an aerospace-specific base oil. The Si–O–Si bonds in POSS groups undergo selective bond cleavage under AO irradiation to generate cage-like POSS silicon oxides, thereby effectively blocking AO erosion of the lubricant, as shown in Figure 3. Furthermore, addressing the lubricant creep problem in space microgravity environments, Bai et al. [37] synthesized a fluorine-functionalized gelator that exploits strong hydrogen bonding interactions with the ether-oxygen segments in perfluoropolyether (PFPE) base oil to construct a highly stable supramolecular oleogel network. This gel system demonstrated exceptional anti-creep characteristics: even under harsh conditions such as prolonged AO or intense ultraviolet irradiation, the stable gel network effectively suppresses thermal capillary migration of PFPE, ensuring long-term retention of the lubricant at the friction interface. The aforementioned studies demonstrate that through functional modification of gelators, resistance to AO erosion and anti-creep performance can be achieved, providing structural solutions for the design of long-life aerospace lubricating materials.

**Figure 4:**
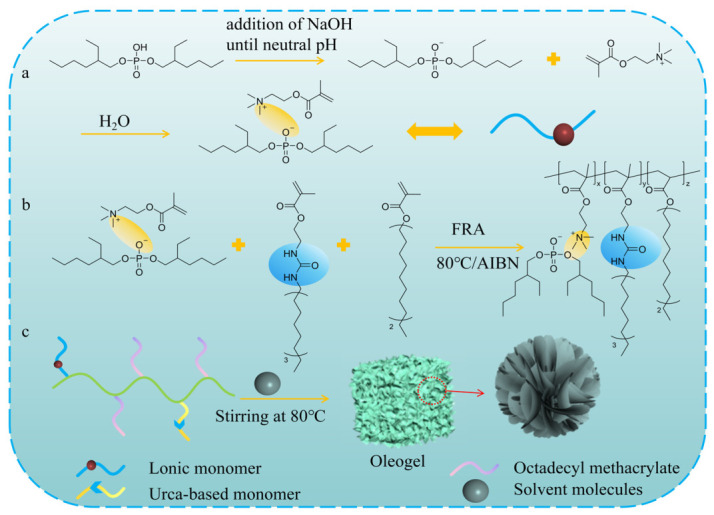
(**a**) Schematic diagram of the synthesis of the MDEHP IL monomer, (**b**) synthesis diagram of the PMUS-P gelator, and (**c**) preparation and gelation diagram of the PMUS-P oleogel.

For extreme operating conditions involving high loads, supramolecular networks constructed by single low-molecular-weight gelators still exhibit deficiencies in structural strength. Although low-molecular-weight gelators exhibit high storage moduli, their yield stresses are relatively low. Once the shear strain at the friction interface exceeds their limit, the gel network is prone to structural collapse and being squeezed out of the friction region, exhibiting structural brittleness. Flexible gel networks possessing high storage modulus and high yield stress can maintain their three-dimensional structural integrity even under extreme operating conditions, thereby firmly locking the base oil to form an exceptionally thick and continuous boundary lubricating film on the surfaces of the friction pair, effectively preventing direct asperity contact between metal surfaces [38]. To further enhance mechanical strength, researchers have developed two representative strategies centered on network structure reinforcement: polymer backbone reinforcement and dual-component synergistic assembly. These aim to construct high-strength and robust gel networks by introducing long-chain entanglement or enhancing intermolecular forces to cope with severe friction conditions. Wang et al. [39] developed a urea-functionalized polymer-based gelator (PUMA-PSMA), where the covalent backbone provides primary structural support while urea side chains construct hydrogen bonding networks. The synergistic effect of covalent and non-covalent bonds enhances the binding capacity of the gel for base oil, forming a denser and more stable network structure compared to monomer gels, thereby significantly improving mechanical strength. This synergistic effect enables the system of storage modulus to reach approximately 10^4.5^ Pa, and the yield stress is also significantly increased to 700 Pa, as shown in Table 1. At the tribological interface, this robust network with high yield strength effectively resists high contact stress. Similarly, Zhang et al. [40] synthesized a polymer gelator through copolymerization of urea monomers with octadecyl methacrylate, which self-assembles in PAO10 to form a high-strength gel network. Compared to monomeric gels, under a load of 200 N, the polymer gel drastically increases the yield stress to nearly 800 Pa. This highly flexible network structure provides a more durable foundation for structural support. Furthermore, Wang et al. [41] revealed the decisive influence of polymer microscopic packing morphology on mechanical properties. The phosphorus-containing anionic polymer xerogel (the state after washing away the base oil from the gel) designed by their group exhibits a porous nanoflower structure with stacked layers, as shown in Figure 4. The gelator molecules self-assemble into sheet-like structures through non-covalent interactions, which further construct a three-dimensional porous network through layered stacking. This layered porous nanoflower structure can effectively disperse applied loads under stress, substantially enhancing the structural stability of the network. Compared with the 500SN base oil, the friction coefficient is reduced by approximately 41.18%. Notably, polymer-based supramolecular oleogel lubricants not only exhibit improved mechanical strength but also, compared to low-molecular-weight gelators, possess longer polymer chain lengths that more readily spread at friction interfaces, forming thicker and more continuous adsorbed oil film layers. This effectively increases oil film thickness under lubrication conditions, delays the occurrence of direct metal contact, and substantially enhances tribological performance. In the dual-component synergistic self-assembly strategy, gel structures can also be reinforced by constructing multi-interaction networks including ion pairs, hydrogen bond complexation, and hydrophobic interactions. Zhang et al. [42] constructed a supramolecular oleogel lubricant (OSW oleogel) using sodium dodecyl sulfate (SDS) and oleic acid (OA) in white oil (WO). The gelation mechanism originates from the sodium carboxylate ion pairs formed between OA and SDS, accompanied by hydrogen bonding and van der Waals interactions that synergistically drive network assembly, forming self-helical fiber structures with flexible entanglement characteristics, thereby enhancing mechanical strength. Ding et al. [43] further elucidated the relationship between alkyl chains and network strength through comparative studies with OSW oleogel. After replacing OA with stearic acid (SA), the introduction of long-chain saturated fatty acids enhanced van der Waals interactions and promoted ordered close packing of molecules, thereby constructing a more robust three-dimensional network. Conversely, the chain bending caused by the double bond in OA weakens this packing effect. Zhang et al. [44] employed a dual-component system composed of cholesterol (CHOL) and bis(2-ethylhexyl) sulfosuccinate (AOT), similarly validating the universality of multi-component synergistic effects in enhancing gel structural strength. Compared to the other two dual-component systems, the yield stress is significantly increased to 400 Pa, as shown in Table 1. This is likely because OA and SA are essentially flexible aliphatic single chains, which are prone to deformation and yielding under extreme high-pressure contacts. In contrast, CHOL possesses a rigid steroid ring structure, acting as the primary support in the network similar to a polymer backbone. Meanwhile, the dual-branched hydrophobic tail chains of AOT wrap around the rigid rings of CHOL, forming a dense gel network. In summary, among network reinforcement strategies for coping with high-load extreme conditions, polymer backbone reinforcement and dual-component synergistic assembly exhibit distinctly different rheological characteristics and lubrication behaviors. Polymer-based supramolecular oleogel lubricants possess an overwhelming advantage in mechanical strength. Even when the limit of dual-component systems is pushed to approximately 400 Pa by introducing CHOL with a rigid steroid ring structure, they still cannot rival polymer systems. This massive discrepancy in strength originates from the different natures of network crosslinking. Dual-component systems rely entirely on the physical stacking of non-covalent bonds (such as hydrogen bonds and ion pairs), and these physical crosslinking points are easily destroyed under extreme shear forces. Conversely, polymer types rely on robust covalent main chains and physical entanglement between long chains, forming an extremely high mechanical yield energy barrier. However, although polymer systems have extreme load-bearing capacity, they also have certain limitations. The structural reconstruction speed of polymer systems is slow, far inferior to the rapid healing of small molecules. Future research should be dedicated to developing smart-responsive supramolecular polymer oleogel lubricants, such as introducing dynamic reversible disulfide bonds to endow them with intelligent responsive healing capabilities. Furthermore, although current research on supramolecular oleogels mainly focuses on material synthesis and basic rheological/tribological testing, cross-scale theoretical modeling and simulation are indispensable to truly apply them in complex engineering mechanical systems. For instance, when facing complex three-dimensional non-uniform line contact conditions such as gears or harmonic drives, advanced elastohydrodynamic lubrication (EHL) models combined with spatial modification optimization methods can accurately predict the impact of complex geometric deformations on oil film pressure distribution and load-carrying capacity [45]. Meanwhile, under transient mixed EHL conditions, employing nonlinear dynamic models to evaluate the dynamic response of lubricants has been proven to be key to improving prediction accuracy [46]. In the future, combining these macroscopic complex contact mechanics models with supramolecular oleogels to develop dedicated transient dynamics prediction algorithms for oleogel lubricants will be the inevitable path to breaking the barrier between advanced lubricating materials and industrial applications.

**Figure 5:**
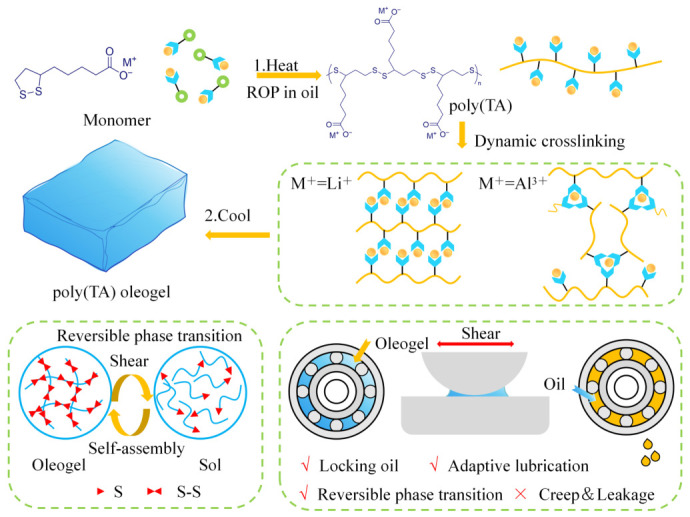
Schematic illustration of fabrication of poly(TA) oleogels with mechanical force-induced reversible phase transition and their application as a self-adaptive lubricant.

To address the unpredictability of extreme operating conditions, developing intelligent supramolecular oleogel lubricating materials with environmental sensing and structural response capabilities represents a frontier direction in the field of tribology. Such lubricants can dynamically adjust their rheological states in response to external stimuli (shear, heat, light, etc.), achieving on-demand lubrication and self-repair, which is significant for ensuring the long-term stable operation of mechanical systems. Xie et al. [47] constructed a dynamic covalent network containing disulfide bonds through ring-opening polymerization of lipoic acid, preparing a class of mechanically responsive and self-adaptive supramolecular oleogel lubricants, as shown in Figure 5. This system utilizes the cleavage and restoration of disulfide bonds under shear to achieve rapid reversible transitions from gel state to sol state lubrication. This strategy elevates the gel-sol phase transition mechanism to the level of dynamic reversible covalent bond regulation, with structural recovery time requiring only a few seconds while maintaining stable rheological response through multiple loading-unloading cycles. This performance significantly outperforms traditional shear-thinning systems that rely on weak interactions, thereby enhancing the service reliability of intelligent lubrication systems under dynamic operating conditions. Wang et al. [48] focused on intelligent continuous repair of interfacial oil films and developed a small-molecule gelator derived from methionine. Molecular dynamics simulations demonstrated that the highly dynamic hydrogen bonding network can drive rapid reorganization of gel molecules at the moment of oil film rupture, thereby maintaining the continuity of the interfacial lubricating layer. Further research revealed that this self-healing behavior exhibits dependence on base oil polarity: base oils with higher aromatic content competitively disrupt the interactions between gelator molecules, thereby weakening network reconstruction efficiency. This indicates that the realization of intelligent self-healing lubrication requires balancing oil-gel and gel-gel interactions. Furthermore, infrared light-responsive functionality has further expanded the applications of supramolecular oleogels. Cui et al. [49] introduced MXene@mGLM composite functional materials into a supramolecular oleogel constructed from 12-hydroxystearic acid, achieving synergistic integration of lubrication and photothermal response functionality. The near-infrared absorption and photothermal conversion capability of MXene in the composite system, combined with the high thermal conductivity characteristics of liquid metal mGLM, accelerates local heat transfer and dissipation, enabling the oleogel to achieve reversible gel-sol transitions under light irradiation, thereby endowing the lubrication system with controllable response capability. Ruan et al. [50] introduced supramolecular oleogels into macroporous polyimide matrices, constructing a recyclable intelligent lubrication system featuring external field-triggered release, capillary recovery, and gel re-locking of oil. This composite structure can release sol-state lubricant under thermal or mechanical stimulation to participate in interfacial lubrication, while upon removal of external fields, the lubricant is re-absorbed through capillary action and restored to gel state, achieving closed-loop utilization and efficient retention of lubricant (oil retention rate of approximately 99%). This strategy provides a feasible pathway for addressing lubricant consumption and replenishment issues under complex operating conditions. Whether in terms of radiation resistance and anti-creep characteristics, strength enhancement under high-load conditions, or intelligent lubrication, functional design has endowed lubricants with more reliable performance under extreme operating conditions. Particularly in addressing lubrication demands under dynamic environments, supramolecular oleogel lubricants have demonstrated enhanced functional potential through responsive regulation mechanisms such as mechanical response and self-healing capabilities, driving innovative breakthroughs in lubrication technology under extreme conditions.

## 4. Nanocomposite Supramolecular Oleogel Lubricants

Nanomaterials, owing to their outstanding extreme-pressure, anti-wear, and friction-reducing properties, are regarded as key additives for enhancing lubricant performance. However, due to their extremely high specific surface areas and surface energies, nanomaterials remain highly unstable in traditional liquid lubricants. They are highly prone to agglomeration and sedimentation driven by strong van der Waals forces. This dispersion stability issue has become a core bottleneck severely restricting the application of nanomaterials in the lubrication field. The most commonly used modification-based dispersion mechanism primarily relies on solvation and steric hindrance effects to enhance the compatibility between nanomaterials and base oils, forming a physical barrier on the nanomaterial surfaces to prevent their approach. However, this mechanism has inherent limitations in pure liquid systems. The Brownian motion of nanomaterials may overcome the steric hindrance, leading to secondary agglomeration, followed by gravity-dominated sedimentation. Therefore, traditional dispersion strategies in pure oil systems can often only temporarily delay, rather than achieve, long-term dispersion stability.

In recent years, constructing three-dimensional networks via the self-assembly of supramolecular oleogels has emerged as a novel strategy to overcome the long-term dispersion stability challenges of nanomaterials in oil phases. The three-dimensional network of supramolecular gels acts as a dense cage, encapsulating nanomaterials in situ and establishing a spatial confinement dispersion mechanism. By utilizing densely entangled gel fibers, this confinement mechanism physically interrupts the close contact and collision between nanomaterials, thereby suppressing agglomeration. Furthermore, the solid-like behavior of supramolecular oleogels in the static state provides sufficient yield stress to resist the gravitational sedimentation of nanomaterials. Currently, various nanocomposite supramolecular oleogel lubricants have been developed. Their dispersion mechanisms have evolved from simple physical confinement within the gel network to more effective interfacial interactions between the nanomaterials and the gel matrix.

The most fundamental dispersion strategy relies on the physical confinement mechanism of the gel network, which effectively inhibits the migration, sedimentation, and agglomeration of nanomaterials. Based on this mechanism, Bai et al. [51] gelated perfluoropolyether (PFPE) base oil using bisamide small-molecule gelators and introduced MoS_2_ to prepare nanocomposite supramolecular oleogel lubricants. Inverted vial tests revealed neither PFPE outflow nor nanoparticle stratification, indicating that gelation significantly enhanced the dispersion stability of the system. Zhang et al. [52,53] developed a novel semi-solid sub-nanowire (SNW) superlubrication material, and the prepared SNWs-PAO6 nanocomposite supramolecular oleogel lubricant exhibited excellent tribological performance. Studies demonstrated that the three-dimensional confined network structure can maintain the dispersion stability of nanomaterials under macroscopic disturbances (centrifugation, low temperature, shear). Zhang et al. [54] designed Ag@DT-COF nanocomposite materials by embedding Ag nanoparticles into the porous channels of covalent organic frameworks (DT-COF), and combined with 12-hydroxystearic acid gelator to prepare nanocomposite supramolecular oleogel lubricants in base oil. The supramolecular gel network restricted the movement of composite nanomaterials in the oil phase, while Ag@DT-COF further enhanced the tribological performance of the lubricant. To elucidate the microscopic mechanism of supramolecular gel networks on nanomaterial dispersion stability, Bai et al. [55] introduced various nanoparticles (Ag, SiO_2_, CaCO_3_, MoS_2_, CF) into base oil PAO10 and prepared different nanoparticle composite supramolecular oleogel lubricants through self-assembly of low-molecular-weight gelators (LMWG) under hydrogen bonding and van der Waals interactions. Combined with molecular dynamics simulations, they systematically analyzed the interaction behavior among gelator molecules, nanoparticles, and base oil molecules. From a kinetic perspective, simulation results demonstrated that when nanoparticles are situated within supramolecular gel networks, the growth rate of their mean square displacement over time is significantly reduced, indicating that the effective diffusion coefficient of particles is notably smaller than in non-gelated systems. This result indicates that the presence of the gel network significantly restricts the long-range migration capability of nanoparticles, making it difficult for them to undergo large-scale displacement within the system, thereby suppressing nanoparticle sedimentation and agglomeration processes. Further radial distribution function analysis revealed the intrinsic reasons for the gel network weakening nanoparticle agglomeration tendency. In non-gelated systems, nanoparticles exhibit pronounced correlation peaks at short-range distances, reflecting a strong aggregation tendency between particles. In supramolecular gel systems, these correlation peaks are significantly weakened or even disappear, indicating that the presence of gelator molecules and their self-assembled structures introduces effective spatial and energy barriers that inhibit close-range particle contact, thereby reducing the probability of particles undergoing close-range contact. This study elucidated the microscopic mechanism by which gel network confinement enhances nanoparticle dispersion stability from a kinetic perspective. This strategy breaks through the key bottleneck of difficult stable dispersion of nanomaterials in traditional lubrication systems, enabling nanoparticles to exist uniformly in the system for extended periods and fully exploit their functional advantages in friction reduction, wear resistance, and thermal conductivity. This provides a reliable carrier for the efficient application of nanomaterials in the lubrication field and offers new design concepts for constructing high-performance, multifunctional lubrication systems.

**Figure 6:**
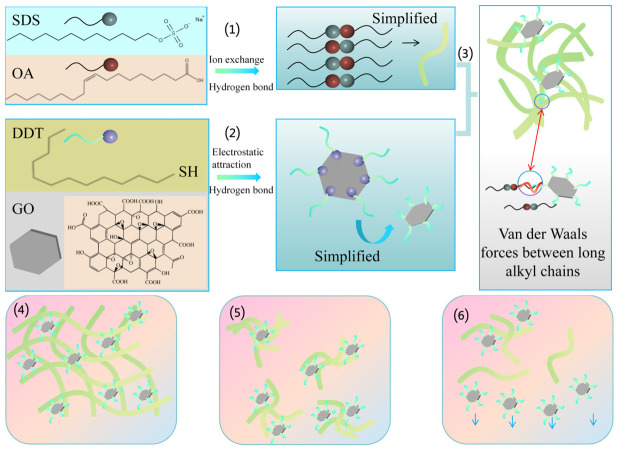
Dispersion mechanism of GO/DDT dragged by SDS-OA fibers in WO: (1) formation of a single micelle, (2) GO modified with DDT, and (3) SDS-OA network stabilizing GO/DDT. Dispersion states when (4) SDS-OA concentration is above CGC, (5) SDS-OA concentration is slightly below CGC, and (6) SDS-OA concentration is too low.

To further enhance dispersion stability, researchers have employed surface modification to construct a non-covalent interfacial coupling mechanism between nanomaterials and the gel network. By grafting alkyl or polymer chains onto the nanomaterial surfaces, a dense protective layer is formed. This not only significantly improves their compatibility in non-polar oil phases, but the outwardly extending chains also provide a strong steric hindrance effect, effectively preventing secondary agglomeration. Moreover, within the supramolecular oleogel system, these surface chains can entangle with the gel network via non-covalent interactions (e.g., van der Waals forces, hydrophobic interactions, and hydrogen bonding). Consequently, building upon the initial spatial confinement, the nanomaterials are further anchored into the gel matrix, achieving long-term dispersion stability. Xia et al. [56] modified graphene oxide (GO) using dodecanethiol (DDT). Driven by hydrogen bonding and electrostatic interactions, DDT introduces flexible long alkyl chains onto the GO surface, which weaken the π–π stacking interactions between GO sheets and prevent agglomeration. Simultaneously, strong van der Waals forces between the alkyl chains on the GO surface and those of the gel network effectively drag and disperse the nanomaterials, confining them stably within the network, as shown in Figure 6. This high degree of interfacial interaction enables excellent dispersion stability for up to one year. Similarly, Guo et al. [57] utilized the −COOH and −OH groups of the 12-HSA gelator to self-assemble a gel network via hydrogen bonding and van der Waals forces, effectively capturing the base oil and Ti3C2TxMXene nanomaterials. By modifying the Ti3C2TxMXene with octadecylphosphonic acid, its compatibility with the base oil was significantly enhanced. This surface alkylation also weakened the mutual attraction between the MXene sheets, allowing the hydrophobic tail chains of the gelator to entangle with them through hydrophobic interactions. Furthermore, researchers have also utilized ionic liquids (ILs) for nanomaterial modification. Gan et al. [58] synthesized phosphonium organophosphate ILs and utilized them to modify graphene, constructing nanocomposite supramolecular oleogel lubricants in non-polar hydrocarbon-based oils. Long alkane chains adsorb onto graphene surfaces under van der Waals interactions to form an adsorption layer, effectively reducing agglomeration between nanomaterials and maintaining good dispersion states. This strategy demonstrates tremendous application prospects. Currently, most ILs are virtually insoluble in non-polar hydrocarbon-based oils, while non-polar hydrocarbon-based oils occupy a significant position in the lubricant market. The ILs synthesized by Gan et al. [58] exhibit excellent solubility in non-polar hydrocarbon-based oils, providing a new pathway for developing non-polar oil-soluble IL-nanocomposite supramolecular oleogel lubricants. Compared to small-molecule surface modification, some researchers choose to graft polymers onto nanomaterial surfaces. The dense long polymer chains can provide a powerful steric hindrance effect far exceeding that of small molecules. Qian et al. [59] in situ grafted poly(lauryl methacrylate) (PLMA) polymer brushes onto the surface of functionalized MOF (UiO-67) nanoparticles. These grafted long polymer chains form an extremely thick and flexible spatial protective barrier outside the nanoparticles, generating a strong steric hindrance effect. Simultaneously, these highly extended outward chains can undergo deeper non-covalent entanglement with the gel network, thereby substantially enhancing the long-term dispersion stability of the nanoparticles. Regarding non-covalent interactions, the synergistic assembly of amphiphilic polymer gelators also demonstrates unique advantages. Liang et al. [60] prepared various nanocomposite supramolecular oleogel lubricants using amphiphilic telechelic polymer gelators, achieving uniform dispersion of nanomaterials by exploiting the compatibility between lipophilic segments and base oils as well as hydrogen bonding interactions between oil-insoluble segments and GO, h-BN, and g-C_3_N_4_ nanomaterials.

**Figure 7:**
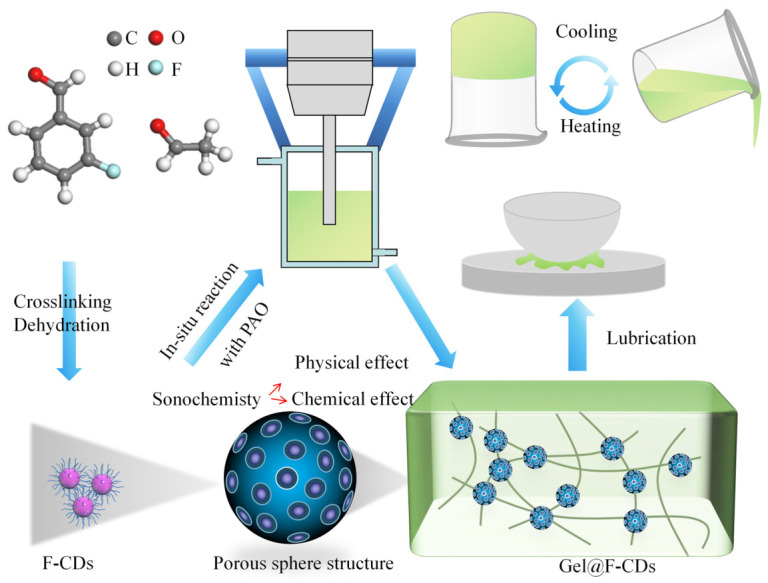
Preparation of F-CDs and Gel@F-CDs.

Distinct from the non-covalent interfacial coupling mechanism, some researchers have advanced the dispersion mechanism to the highest-dimensional gel network nodes integration. This mechanism completely subverts the conventional concept of treating nanomaterials merely as passive inclusions. By endowing nanomaterials with self-assembly capabilities or constructing dynamic chemical/coordination bonds between the nanomaterials and the gel matrix, nanomaterials deeply participate in network construction, transforming into active cross-linking nodes within the gel network. This mechanism represents the most cutting-edge strategy currently available for resolving the long-term dispersion stability issues of nanolubricants. A typical strategy is to endow nanomaterials with self-assembly capabilities. Wang et al. [61] modified liquid metal (GLM) nanoparticles via the self-assembly of dopamine derivatives, and grafted urea-based gelators onto the GLM surface through free radical polymerization to prepare Gelator@GLM. During the gel formation process, GLM is no longer passively confined within the gel network, but acts as a network node, deeply participating in the self-assembly construction of the supramolecular oleogel network. Jin et al. [62] designed a class of fluorine-doped carbon dots (F-CDs) as gelators to prepare nanocomposite supramolecular oleogel lubricants in PAO base oil, as shown in Figure 7. The fluorine-doped carbon dots transformed from conventional lubricant additives into building units of the gel network, fundamentally avoiding subsequent dispersion and agglomeration issues of nanomaterials. Similarly, Zhang et al. [63] decorated bisurea compounds on h-BN nanosheet surfaces, introducing the self-assembly capability of small-molecule gelators into nanomaterials and endowing them with gelation ability, thereby improving the dispersion state of nanosheets. This strategy ensures uniform distribution of nanomaterials at the friction interface, greatly enhancing tribological performance. When friction occurs, the gel transforms to sol under stress, and nanomaterials spread along the shear direction to form a perfect protective layer. Once friction ceases, the structure recovers, and nanomaterials are locked again, effectively reducing sedimentation and agglomeration behavior of nanomaterials. Another key strategy is to utilize polymer gel factors to construct strong dynamic chemical bonds and coordination bonds. Dynamic crosslinking strategies based on sulfur chemistry have been applied to the stable dispersion of metal and metal sulfide nanoparticles. Wang et al. [64] utilized the ring-opening polymerization characteristics of α-lipoic acid esters to prepare nanocomposite supramolecular oleogel lubricants containing dynamic disulfide bonds. Alkyl thiol-modified liquid metal nanodroplets (C12@GLM) are not merely additives but form dynamic disulfide bonds through dynamic covalent exchange between thiol groups on their surfaces and other thiol groups or sulfur atoms on the gel backbone. These bonds can be broken and reconnected, firmly combining GLM with the gel network as covalent crosslinking points, achieving dispersion stability. Chen et al. [65] further extended this concept by designing a telechelic polymer gelator containing 1,2-dithiolane structures. The polymer end groups can form strong coordination interactions with the surfaces of various nanoparticles such as MoS_2_ and WS_2_, firmly anchoring particles within the gel network. Their diffusion and agglomeration behavior are significantly suppressed, enabling the system to maintain dispersion stability for up to one year. Furthermore, Li et al. [66] developed a nanocomposite supramolecular oleogel lubricant that ingeniously exploits the specific binding between sulfur atoms in α-lipoic acid ester triblock polymers and sulfur vacancies on MoS_2_ nanoparticle surfaces, significantly enhancing the dispersion stability of two-dimensional materials. Beyond sulfur chemistry, dynamic covalent bonds based on boronate esters have also been proven to be effective means for enhancing nanomaterial dispersion stability. Xue et al. [67] proposed a strategy utilizing reversible B–O bond formation between phenylboronate ester groups and hydroxyl groups on SiO_2_ or TiO_2_ surfaces to prepare nanocomposite supramolecular oleogel lubricants. These dynamic covalent bonds establish reversible chemical bridges between polymers and nanoparticles, which, combined with the steric hindrance effect of polymer chains, block particle agglomeration pathways. In summary, the dispersion stability mechanisms of nanocomposite supramolecular oleogel lubricants are progressively evolving from single-factor regulation to multi-scale network synergistic regulation. These progressively advancing strategies successfully achieve the long-term dispersion of nano-additives, providing a clear pathway to overcome the dispersion stability bottleneck of nanolubricants.

## 5. Practical Applications and Development of Supramolecular Oleogel Lubricants in Engineering Fields

**Figure 8:**
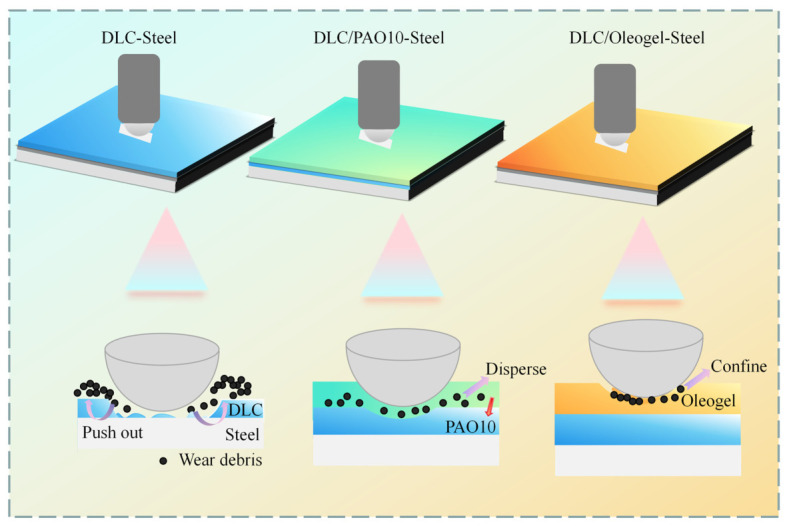
Schematics of mechanism under DLC lubrication, DLC–PAO10 composite lubrication and DLC–HTG composite lubrication.

Although supramolecular oleogels have achieved significant progress in molecular design and laboratory-level performance regulation, their practical applications have rarely been explored. This chapter no longer confines itself to elucidating the properties of oleogel materials themselves but focuses on the engineering applications of supramolecular oleogel lubricants in mechanical systems and special service environments. Wang et al. [68] reported a self-constraining supramolecular oleogel lubricant (HTG) with high phase transition temperature. HTG exhibits excellent creep recovery and thixotropy, with a phase transition temperature reaching 190 °C, making it suitable for porous iron-based and polyamino bearing materials under high-temperature and high-speed environments, extending bearing service life and enhancing bearing oil storage capacity. This lubricant also demonstrates promising application prospects in special mechanical components where sealing is difficult. Furthermore, the failure factors of modern critical mechanical components such as bearing parts, electric vehicles, and wind power equipment components involve not only friction and wear issues, but electrical corrosion caused by the insulating properties of lubricating oil films is also a key factor. Liu et al. [69] developed a supramolecular oleogel lubricant using CuS@MXene as a multifunctional additive, effectively enhancing lubrication performance and reducing the electrical resistance of lubricating films, providing an effective approach for suppressing electrical corrosion in mechanical systems. Beyond the singular use of supramolecular oleogel lubricants, researchers have combined them with solid lubricating coating materials to develop novel lubrication systems. Yu et al. [70] constructed a diamond-like carbon thin film (DLC)-supramolecular gel (HTG) composite lubrication system, which exhibited excellent friction-reducing performance and ultra-low wear behavior even under maximum contact stress as high as 2.24 GPa, as shown in Figure 8. While DLC provides a highly wear-resistant foundation for the system, the HTG reported by Wang et al. [68] enhances overall lubricity and long-term service life through further lubrication, confinement of coating debris, and base oil leakage prevention. Although composite systems demonstrate potential advantages, achieving uniform and stable thin film coatings of prepared DLC on large-sized components (such as wind turbine bearing surfaces) still presents process difficulties, and the relatively high cost of HTG also requires further optimization. Similarly, Zhang et al. [71] designed a MoS_2_ thin film coating and supramolecular oleogel composite lubrication system, likewise achieving improvements in lubrication and service life. Their oleogel lubricant exhibited anti-creep properties, and this system can be applied to aerospace space lubrication. The WS_2_ thin film coating and supramolecular oleogel composite system based on aerospace-specific oil MAC developed by Ye et al. [72] is similarly applicable to space service environments. Furthermore, porous coatings are also highly suitable for combination with supramolecular oleogel lubricants. Liu et al. [73] constructed a porous micro-arc oxidation (MAO) wear-resistant layer on titanium alloy surfaces and introduced supramolecular oleogel lubricants into its porous structure to form a composite lubrication system. While utilizing the friction-reducing properties of MAO, the oleogel achieves long-term locking and load-triggered release of lubricant within the pore channels, thereby continuously replenishing lubrication at the friction interface. Although solid thin films possess excellent friction-reducing characteristics, they are prone to wear depletion and difficult to maintain in long-term service. Under the synergistic effect of supramolecular oleogel lubricants, direct dry friction of solid coatings is avoided, wear debris generation during the running-in period is reduced, and the thixotropy of oleogels prevents oil leakage, further enhancing lubrication. While this composite system improves lubrication, the influence of tribochemical reactions must also be considered. Gelator molecules, base oils, additives, and solid film elements may undergo complex chemical reactions under friction induction. Such reactions may generate beneficial tribofilms or may lead to destruction of gel structures or corrosion of coatings. The mechanisms in this aspect have not yet been investigated. Overall, the lubrication strategy combining solid thin film coatings with supramolecular oleogel lubricants has gradually developed into a generalizable engineering paradigm, effectively breaking through the performance bottlenecks of traditional single lubrication media.

Beyond enhancing lubrication performance in mechanical systems, supramolecular oleogel lubricants also possess application potential in drag reduction and anti-fouling. Parbat et al. [74] constructed a covalently crosslinked biphasic supramolecular oleogel lubricant, achieving long-term stable lubrication under strong shear flow and complex marine environments. The lubricant maintained excellent lubrication and anti-fouling performance after experiencing physical wear, chemical contamination, and high-low temperature cycling, and no obvious fouling attachment was observed during continuous service exceeding two months in real marine environments. Similarly, Lee et al. [75] constructed a supramolecular oleogel based on solid fatty acid amide (FAA), exhibiting excellent drag reduction and anti-biofouling performance. Ice layer accumulation on solid surfaces represents a significant problem for infrastructure, severely affecting its performance. Addressing this engineering challenge, Jasmine et al. [76] constructed a fluorinated organic supramolecular oleogel (F-ORG) that achieves release and reabsorption through temperature changes. When below the critical temperature, the lubricant is released, maintaining surface ice adhesion strength at ≤10 kPa, thereby enabling passive removal of surface ice layers through natural forces such as gravity, wind, and vibration. When above the critical temperature, lubricant absorption can minimize losses from evaporation and discharge, extending its durability. Supramolecular oleogel lubricants have not only demonstrated significant advantages in lubrication performance but have also exhibited tremendous application potential in drag reduction and anti-fouling applications. In the future, with continuous optimization of material design and preparation processes, supramolecular oleogel lubricants are expected to play greater roles in more fields.

## 6. Conclusions

Supramolecular oleogel lubricants construct three-dimensional network structures through non-covalent interactions, achieving effective confinement and interfacial regulation of base oils and functional components while balancing lubricating fluidity and structural stability. Existing research indicates that gelator design and nanomaterial additive incorporation constitute the core themes for performance enhancement of supramolecular oleogel lubrication systems. Through molecular structure design of gelators and regulation of assembly modes, green performance can be imparted, three-dimensional network structures can be strengthened, tribological performance can be enhanced, and intelligent lubrication can be achieved. The introduction of nanomaterials to construct composite networks enables long-term stable dispersion of nano-additives under gel confinement effects, allowing their friction-reducing and anti-wear functions to be continuously exerted. Although an increasing number of supramolecular oleogel lubricants have been developed, supramolecular oleogel lubricating materials still face several challenges that need to be addressed:

(1) At present, advanced supramolecular oleogel lubricants face the problem of high cost. Although classic low-molecular-weight gelators like 12-HSA are relatively cheap and highly mature in the industry, the specially designed custom gelators in recent studies have high costs in raw materials and synthesis conditions, which is a key factor facing commercial promotion. Therefore, the commercialization strategy for these materials should be carried out in stages. In the short to medium term, they can target high-value niche markets, fully utilizing their outstanding performance in fields such as aerospace and space environments, where the excellent performance is sufficient to compensate for the high initial cost of custom gelators. In the long run, natural raw material gelators or classic low-molecular-weight gelators can be promoted for the mass market.

(2) Currently, intelligent supramolecular oleogels are largely limited to passive mechanical shear or basic photothermal responses, lacking active tunability under complex engineering conditions. In the future, they can be developed towards magnetic, electrical, and acoustic triggering mechanisms. In terms of magnetic response, by adding functionalized magnetic nanoparticles into supramolecular oleogels, the lubricant exhibits magnetorheological behavior. This realizes the active, on-demand, and directional delivery of the lubricant to the friction contact zone, but its limitation lies in preventing the agglomeration of rigid nanoparticles, which requires detailed research in the future; in terms of electrical response, conductive materials can be introduced to enable the network to undergo electrorheological transitions. This mechanism only requires adjusting the external voltage to achieve instantaneous modulation of the lubricant state, which is highly promising in microelectromechanical systems, but electrical stress may cause irreversible electrochemical degradation or anodic oxidation of base oils and gelator molecules; in terms of acoustic response, high-frequency ultrasound can be used to selectively induce the reversible cleavage of non-covalent bonds. Acoustic triggering can penetrate deep structural barriers without direct contact, promoting a rapid local gel-sol transition to achieve the precise release of base oil for lubrication. In addition, at the design level of intelligent supramolecular oleogels in the future, chromogenic sensing groups can be introduced to judge the friction state through the color of the lubricant.

(3) Current supramolecular oleogel lubricants focus predominantly on gelator design while neglecting the regulatory role of base oils on gel network structure and interfacial behavior. Existing research has demonstrated that base oil viscosity, molecular structure composition (such as branched alkane proportion and aromatic content), and fatty acid composition of vegetable oils all influence gel three-dimensional network structure and lubrication performance. Future research should elevate base oils from passive solvents to structural regulation units, conducting systematic studies using base oil molecular structure parameters as variables to establish quantitative correlations among base oil-gel network structure-interfacial lubrication performance, thereby providing guiding design criteria for synergistic matching design of base oils and gelators under different operating conditions [28,31,32].

(4) To promote the widespread application of supramolecular oleogels, corresponding standardization systems need to be established, including synthesis standards for gelators, testing standards for gel performance, and quality standards for products.

(5) Polymer-based supramolecular oleogel materials developed based on dynamic covalent bonds have been proven to have excellent mechanical strength and self-healing properties, but currently, only designs such as disulfide bonds exist. In the future, more advanced dynamic covalent bonds can be introduced to achieve millisecond-level rapid healing performance.

Although the future development of supramolecular oleogel lubricating materials faces numerous challenges, it is undeniable that they have broken through the limitations of conventional lubricants. Their unique advantages make development full of potential. It is believed that supramolecular oleogel lubricants with superior performance will certainly be developed in the future, demonstrating broad application prospects in the lubrication field.

## Figures and Tables

**Table 1:** Representative quantitative values of functionalized supramolecular oleogels for structural reinforcement under extreme conditions.

Gelator	Base Oil	Content	Storage Modulus G’ (pa)	Yield Stress (pa)	Content	COF	Load (N)
Monomer gel	PAO10		≈10^3.5^	≈80	12.5%	≈0.17	200
Polymer gel	PAO10		≈10^4.8^	≈800	17.5%	≈0.15	200
	PAO10					≈0.25	200
PUMA-PSMA	500SN	15%	≈10^4.5^	≈700	15%	≈0.02	30
PMUS-P	500SN	15%	≈10^3^~10^3.5^	10^2^~10^2.5^	15%	≈0.1	300
	500SN					≈0.17	300
OA-SDS	WO	7.2%	≈10^2.5^	14.5	7.2%	≈0.12	100
	WO					≈0.23	100
SA-SDS	WO		≈10^3^	33.22		0.054	
	WO					0.071	
CHOL-AOT	WO	5%	≈10^3.8^	≈400	3%	≈0.11	100~600
	WO					≈0.2	100~600
HMTA	150BS	3%	≈10^3.8^	4.12	3%	0.11	100~700
	150BS					0.14	100~700

## Data Availability

No new data were created or analyzed in this study.
